# Correlation Between Vision and Cognitive Function in the Elderly: A Cross-Sectional Study

**DOI:** 10.1097/MD.0000000000002423

**Published:** 2016-01-22

**Authors:** Oriel Spierer, Naomi Fischer, Adiel Barak, Michael Belkin

**Affiliations:** From the Department of Ophthalmology, Tel Aviv Sourasky Medical Center, Sackler Faculty of Medicine (OS, NF, AB); and Ophthalmic Technologies Laboratory, Goldschleger Eye Research Institute, Tel Aviv University, Tel Hashomer, Israel (MB).

## Abstract

The correlation between vision and cognition is not fully understood. Visual impairment in the elderly has been associated with impaired cognitive function, dementia, and Alzheimer disease. The aim was to study the correlation between near visual acuity (VA), refraction, and cognitive state in an elderly population.

Subjects ≥75 years were enrolled in this cross-sectional study. Refraction and near VA was tested. Cognitive function was evaluated with a version of the mini-mental state examination for the visually impaired (MMSE-blind). The eye with better VA and no cataract or refractive surgery was analyzed.

One-hundred ninety subjects (81.6 ± 5.1 years, 69.5% female) were included. Good VA (≤J3) was associated with high MMSE-blind (>17) (OR = 3.18, 95% CI = 1.57–6.43, *P* = 0.001). This remained significant adjusting for sex, age, and years of education. Wearing reading glasses correlated significantly with high MMSE-blind after adjustment for sex and age (OR = 2.14, 95% CI = 1.16–3.97, *P* = 0.016), but reached borderline significance after adjustment for education. There was a trend toward correlation between myopia and better MMSE-blind (r = −0.123, *P* = 0.09, Pearson correlation).

Good VA and wearing glasses seem to correlate with better cognitive function. Reading glasses can serve as a protective factor against cognitive deterioration associated with sensory (visual) deprivation in old age. The association between myopia and cognition requires further investigation.

## INTRODUCTION

Visual impairment in the elderly has been associated with impaired cognitive function, dementia and Alzheimer disease.^1–5^ Recent findings have shown that subjects with good visual acuity (VA) are at a 63% decreased risk for developing dementia over an 8.5-year period.^2^ Similarly, elderly individuals with decreased VA are 5 times more likely to show diminished cognitive performance compared to elderly individuals with good vision.^2^ In addition, VA was reported to influence memory capability in the elderly population.^3^ The Age-Related Eye Disease Study Research Group suggested a possible association between advanced age-related macular degeneration (AMD) and VA with cognitive impairment in older persons.^4^ Accordingly, it is possible that regular eye checkups followed by appropriate treatment for ocular pathology could decrease the risk for developing dementia or Alzheimer disease.^2^ The hypothesis that there is a correlation between vision and cognition is based on the impact of vision on mentally stimulating activities. Loss of vision hinders the performance of these tasks (eg, reading, socializing) and may result in behavioral changes and cognitive decline. Deterioration in vision reportedly reduces physical, mental, and psychosocial activities which can lead to a poor cognitive state.^6–8^ An alternative hypothesis refutes a causal relationship between VA and cognitive abilities, and claims that both are part of brain aging.^9^

Refractive errors and cognitive impairment are common conditions among older people.^10^ However, there are limited data regarding linkage between them, especially in the elderly. In a study of low birth weight children a correlation was found between near distance visual impairment and low IQ.^11^ The Singapore Malay Eye Study found that myopic elderly are at double the risk of having cognitive impairment as opposed to emmetropes or hyperopes.^12^ The purpose of this work, therefore, was to determine whether there is a correlation between VA, refraction, and cognitive state in an elderly population with no known dementia or Alzheimer disease.

## METHODS

Subjects aged 75 years and older who visited geriatric day care centers were candidates for this cross-sectional study. To obtain a significant sample size, 200 sequential participants were enrolled between July 2012 and October 2012. To address potential bias, exclusion criteria were: a history of bilateral cataract surgery, pathological myopia and any other intraocular or refractive surgical procedure in either eye, illiteracy, or the presence of diagnosed dementia or Alzheimer disease. Informed consent was obtained from all patients. The study adhered to the tenets of the Declaration of Helsinki and was approved by the Ethics Committee of the Tel Aviv Sourasky Medical Center.

A face-to-face interview was conducted, and data on demographics and general health as well as ophthalmic history were recorded. Subjects’ medical records were reviewed to see whether a cataract surgery was performed in one or both eyes. Subjects were also directly questioned whether a cataract surgery was performed in the past. A direct ophthalmoscope was used to verify that the patient was not pseudophakic in the examined eye. Cognitive function was evaluated with the mini-mental state examination.^13^ The mini-mental state examination for the visually impaired (MMSE-blind) was calculated by removing 8 items that involve vision (2 items with naming, following a written command, writing a sentence, copying, and performing a 3-stage command),^14,15^ thus leaving a total possible score of 22 as compare to a score of 30 for the full MMSE. A high MMSE-blind score was defined as >17 and a low MMSE-blind score was defined as ≤17.^14^

Noncycloplegic refraction (sphere, cylinder, and axis) was measured with a portable autorefractometer (Righton Retinomax 3, Right Medical, Virginia Beach, VA). Spherical equivalent refraction was calculated as sphere + half cylinder power. Corrected near VA was tested using the Jaeger chart. A good VA was defined as ≤J3, and a poor VA was defined as >J3. J3 was classified as representing normal functional ability to read print the size of common everyday reading material (such as newspapers).^16^ Only phakic eyes were included in the study. When both eyes were phakic, then the eye with the better VA was used for analysis. If both eyes had the same VA then the right eye was used for the data. If the subject had undergone cataract surgery in one eye but their other phakic eye had the same or better VA, then the phakic eye was included. However, if the phakic eye had a worse VA than the operated pseudophakic eye, then the case was excluded from the study.

### Statistical Analysis

Data were recorded on Microsoft Excel spreadsheets. Chi-square testing and univariate analyses were used to assess associations between good and poor MMSE-blind scores (cognitive function) and ocular parameters, which included good/poor near VA, wearing eyeglasses for far or near and refraction (myopia/hyperopia). Multivariate logistic regression analyses were carried out with the MMSE-blind score as the dependent parameter and with all those variables as independent parameters. Multiple linear regression tests were used to check correlations of MMSE-blind scores and continuous parameters (VA and refraction). In addition, Pearson correlation was used to test correlations between myopia and better MMSE-blind scores. Jaeger Chart scores were converted to LogMAR readings in accordance to the accepted standardization.^17^ All analyses were 2-tailed, and significance was set at the 5% level. Data are presented as means (±SD). Statistical analysis was performed with SPSS software (SPSS, Inc., Chicago, IL).

## RESULTS

One-hundred ninety subjects (mean age 81.6 years, 69.5% females) fulfilled the inclusion criteria and comprised the study group. The right eye was studied in 90 cases (47.4%) and the left eye in 100 (52.6%) cases. One-hundred twenty (63.2%) participants reported wearing reading glasses. The study participants’ demographic characteristics, cognitive and ophthalmic data are summarized in Table 1.

**TABLE 1:**
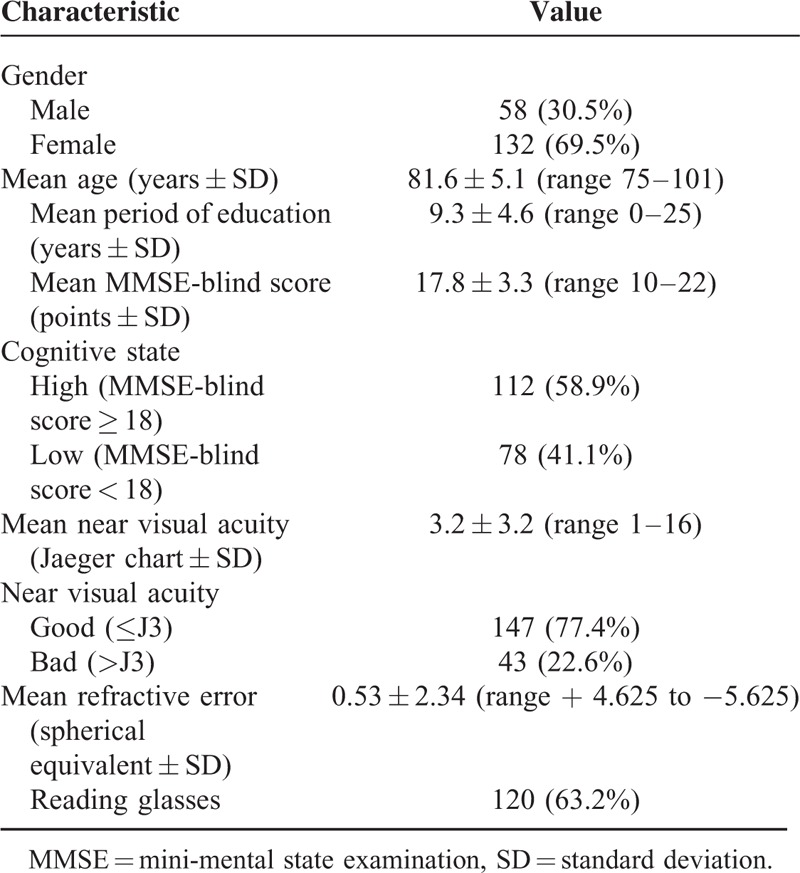
Demographic Characteristics, Cognitive and Ophthalmic Data of 190 Subjects

A higher MMSE-blind score (as a continuous variable) was correlated with better VA (β = −6.0, SE = 0.98, *P* < 0.001, multiple linear regression test) and remained so after adjustment for sex, age, and education (β = −4.32, SE = 0.97, *P* < 0.001, adjusted R^2^ = 0.276, multiple linear regression test). MMSE-blind score was also correlated with VA as a continuous variable (r = −0.405, *P* < 0.001, Pearson correlation). A high MMSE-blind score (>17) was associated with good VA (J3 or lower) (*P* = 0.001, Chi-square) and wearing reading glasses (*P* = 0.026, Chi-square). The association between a high MMSE-blind score and good VA remained significant after adjustment for sex, age, and years of education (*P* = 0.026, multivariate logistic regression model, Table 2). The association between a high MMSE-blind score and wearing reading glasses remained significant after adjustment for sex and age (OR = 2.14, 95% CI = 1.16–3.97, *P* = 0.016, multivariate logistic regression model), and became borderline after adjustment for sex, age, and years of education (Table 3).

**TABLE 2:**
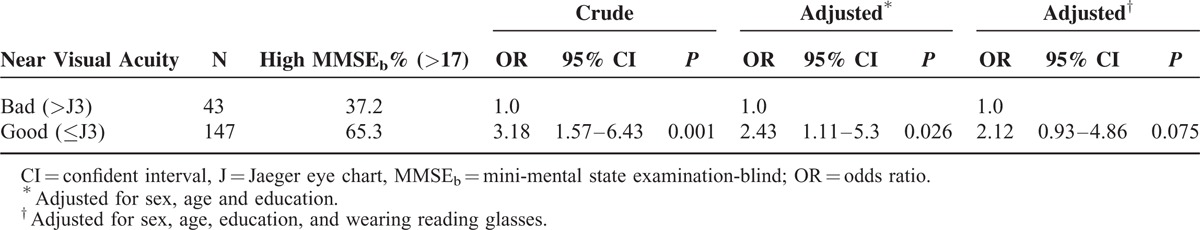
Association Between Visual Acuity and High Mini-Mental State Examination-Blind Score for 190 Subjects

**TABLE 3:**
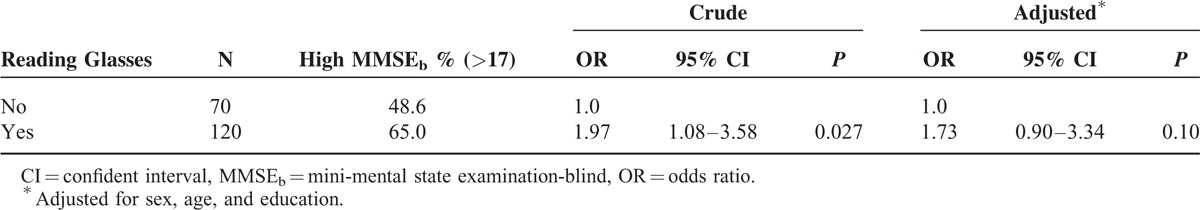
Association Between Wearing Reading Glasses and High Mini-Mental State Examination-Blind Score for 190 Subjects

There was a trend toward a correlation between myopia and a better MMSE-blind score (r = −0.123, *P* = 0.09, Pearson correlation), but it did not persist after adjusting for sex, age, or education (β = −0.07, SE = 0.05, *P* = 0.2, multivariate linear regression model). Younger age and more years of education were correlated with a better MMSE-blind score. An MMSE-blind score >17 was negatively associated with age and positively associated with education (OR = 0.92, 95% CI = 0.87–0.98 for an 1-year increase in age and OR = 1.22, 95% CI = 1.12–1.32 for an 1-year increase in education). Gender was not associated with the MMSE-blind score (*P* = 0.9).

## DISCUSSION

The results of the present study demonstrated that good near VA was significantly correlated with a better MMSE-blind score, reflecting better global cognitive function. Wearing reading glasses was significantly correlated with good cognitive function; however, the linkage was attenuated after adjusting for education. Although there was an association between myopia and better cognitive function, it did not reach a level of significance after adjusting for sex, age, and education. A higher level of education and younger age were also correlated with better cognitive function, whereas gender was not. The correlation between education, age, and cognition was reported in several earlier studies^18,19^ and may provide validation to our study.

The relation between vision and cognition is not fully understood. Specific visual disorders have been shown to share common pathogenic pathways with Alzheimer disease.^2^ Amyloid beta deposition, which is a major extracellular deposit in Alzheimer disease plaques, was found in some AMD eyes, possibly indicative of a common pathophysiology.^20^ The Rotterdam Study showed that subjects with advanced AMD at baseline had an increased risk of incident Alzheimer disease, which may indicate a common pathogenesis for both diseases.^21^ A recent study of age-related eye diseases (AMD, Fuch corneal dystrophy and glaucoma) in patients older than 65, also found a correlation between vision loss and lower cognitive scores.^22^ One hypothesis (“sensory deprivation”) holds that low VA causes reduction in physical and mental activities which, in turn, is a risk factor for declined cognitive function and dementia.^23^ A second hypothesis (“common cause”) is that there is no direct link between VA and intellectual performance but that both are part of brain aging. This approach contends that there is a third factor common to vision and cognition, such as degeneration of central nervous function.^24^ A third theory (“resource allocation hypothesis”) states that individuals with sensory impairment allocate more attention resources to processing sensory information, thus leaving fewer resources for other cognitive tasks.^25^ The association between VA and cognitive function found in the present study is in agreement with a number of studies in this field.^2–5^ Data from the Blue Mountains Eye Study on an Australian population documented a weak but significant cross-sectional correlation between VA and cognitive function in the normal aging population.^26^ A recent report showed that subjects with good VA are at a lower risk for developing dementia over an 8.5-year period.^2^ One study on osteoporotic fractures found a 2-fold increase in odds of cognitive decline over average follow-up of 4.4 years associated with vision impairment in American women.^4^ A recent study by Fong et al^27^ highlighted the importance of studying eye-related problems in vulnerable groups. The Geriatric population was more likely to suffer corneal and scleral perforations due to falls and infections with geriatric nursing home patients displaying a worse prognosis. Thirty-eight percent from these institutionalized patients were found to have dementia.^27^ Reyes-Ortiz et al^28^ concluded that near, but not distance, visual impairment is associated with decreased cognitive function. An association between near vision impairment and cognitive dysfunction was also found in a study of patients with type 2 diabetes.^29^ Several studies, however, have questioned whether there is a causal association between VA and cognitive state.^9,30^

The study hypothesis was that myopic elderly will score higher results in the MMSE than the emmetropic and hyperopic elderly. This assumption was based on several previous findings. First, myopia had been found to be correlated with higher education, which could potentially reduce the risk for cognitive deterioration.^31^ Second, myopes see the world close-up. They reportedly have better ability to narrow their attention to a small space^32^ and perform spatial rotation.^33^ Myopia could therefore be a protective factor against dementia. The third possible mechanism involved in cognitive deterioration is the reduction in visual input to the brain resulting from the development of presbyopia. Many emmetropes postpone the use of reading glasses for near tasks, such as reading, thereby considerably restricting detailed visual input to the brain. Myopes, on the other hand, achieve detailed vision for close tasks by simply removing their eyeglasses. The decreased near visual input over a period of several years among emmetropes in their 40s could influence the cognitive decline occurring years later. Our results showed a correlation of borderline significance (r = −0.123, *P* = 0.09) between myopia and good cognitive function, but it disappeared after adjustment for age, sex, and years of education. At the time we were conducting this research, the Singapore Malay Eye Study group published their results, which are in opposition to ours. They found that myopes had a double risk for cognitive decline as opposed to emmetropes and hyperopes.^12^ Those authors postulated that pathogenic processes in dementia, including amyloid beta and acetylcholine deficiency, may affect refraction. They concluded that their findings are preliminary and need to be further tested.^12^ There are several differences between the present study and the Singapore Malay Eye Study. We examined people aged 75 to 101 years while the age range of their study population was 60 to 79 years. They used the Abbreviated Mental Test (AMT), which is a 10-question test of general cognitive function,^12^ and we used the more commonly administered and extended MMSE. The AMT has been reported as being inferior to MMSE as a screening tool, with MMSE remaining as the best tool for primary care clinicians.^34^ Finally, the differences in study populations might have had a telling impact on the results.

Whether or not vision correction can improve cognitive function in the elderly is controversial issue. Tamura et al^35^ documented improvement in cognitive function in subjects following cataract surgery. Rogers and Langa^2^ found that persons diagnosed with dementia had received fewer ophthalmologic services before their diagnosis than those who aged with normal cognition. They postulated that treatment of eye pathologies may affect the probability of developing dementia, and that under-treatment of visual problems may contribute to cognitive decline.^2^ Elliott et al.,^36^ however, found that vision correction by means of cataract surgery or refractive correction (eyeglasses) did not improve short-term cognitive function. Nevertheless, it is possible that preventing visual impairment could help prevent the development of cognitive decline. It was recently suggested^37,38^ that under-corrected refractive error has a role in developing cognitive dysfunction. This possibility may have important implications because refractive error is easily corrected.^37,38^ We observed that subjects wearing reading glasses did better in cognitive function as measured by the MMSE-blind. It is possible that simple availability of corrective eyeglasses has the potential to decrease the risk for cognitive deterioration; however, more studies are needed to support this statement.

The strengths of our research include the use of standardized protocols for obtaining cognitive evaluations. They all were followed by 1 researcher (O.S.), which is particularly important in cognitive tests which are influenced by the way questions are asked and the time given for answers. Reliability increases when all the examinations are carried out by a single examiner. The MMSE we used to assess cognition is the one most commonly used for this purpose. The full version includes 30 questions and is a practical method for diagnosing and grading the level of dementia. The test examines linguistic, computational, memory, concentration, and orientation functions. In the MMSE-blind version, 8 items involving image processing are deleted which may remove confounding factors.^14,15^ Normal cognitive ability is defined as a test result >17, while a lower score may indicate abnormal cognitive function.^14^ We defined good near VA as ≥J3 (Jaeger chart) since, in practice, objects which necessitate near vision, such as reading a telephone directory or newspapers, are not smaller than J3.^16^ MMSE results may be influenced by the intellectual and educational backgrounds of the examinee.^18,19^ Bearing this in mind, the multivariate analysis in this study was adjusted for years of education, thus neutralizing the education effect on test results.

Our work has some limitations. Because the interviews were carried out at adult day care centers and not at a clinic, we could only check near VA by using the Jaeger chart. We could not precisely measure the far VA due to technical restraints. Our findings, however, are consistent with those previously reported that near visual impairment is associated with decreased cognitive function. In addition, refraction was based on autorefractometer readings and not on subjective refraction. Nevertheless, recent studies reported an agreement between autorefractometer and subjective refraction. Autorefractometers have been proven to show valid and repeatable measures of objective refraction when compared with noncycloplegic subjective refraction.^39,40^ Finally, since this is a cross-sectional study, the temporality of poor vision or refractive error and cognitive dysfunction is not clear-cut, and longitudinal studies would be needed to show a causal relationship.

In conclusion, we documented a cross-sectional correlation between vision and cognitive functions in normal aging. Good VA and wearing reading eyeglasses appear to correlate with better cognitive function in the elderly. It is possible that an inexpensive and simple means such as eyeglasses can serve as a protective factor against cognitive deterioration associated with sensory (visual) deprivation in old age, but further studies are needed to verify it. The association between myopia and cognition also warrants further investigations in order to expand our understanding of the nature of the relationship and possible causality between visual and cognitive functions.
